# Using Natural Language Processing to Describe the Use of an Online Community for Abortion During 2022: Dynamic Topic Modeling Analysis of Reddit Posts

**DOI:** 10.2196/72771

**Published:** 2025-07-09

**Authors:** Elizabeth Pleasants, Ndola Prata, Ushma D Upadhyay, Cassondra Marshall, Coye Cheshire

**Affiliations:** 1 School of Public Health University of California, Berkeley Berkeley, CA United States; 2 Department of Obstetrics and Gynecology University of North Carolina at Chapel Hill Chapel Hill, NC United States; 3 Bixby Center for Population, Health, and Sustainability School of Public Health University of California, Berkeley Berkeley, CA United States; 4 Department of Obstetrics, Gynecology & Reproductive Sciences University of California, San Francisco San Francisco, CA United States; 5 School of Information University of California, Berkeley Berkeley, CA United States

**Keywords:** abortion, online community, Reddit, Dobbs decision, natural language processing, topic modeling, BERTopic

## Abstract

**Background:**

Abortion access in the United States has been in a state of rapid change and increasing restriction since the *Dobbs v Jackson Women’s Health Organization* decision from the US Supreme Court in June 2022. With further constraints on access to abortion since *Dobbs*, the internet and online communities are playing an increasingly important role in people’s abortion trajectories. There is a need for a broader understanding of how online resources are used for abortion and how they may reflect changes in the sociopolitical and legal context of abortion access. Research using online information and leveraging methods to work efficiently with large textual datasets has the potential to accelerate knowledge generation and provide novel insights into changing abortion-related experiences following *Dobbs*, helping address these knowledge gaps.

**Objective:**

This project sought to use natural language processing techniques, specifically topic modeling, to explore the content of posts to 1 online community for abortion (r/abortion) in 2022 and assess how community use changed during that time.

**Methods:**

This analysis described and explored posts shared throughout 2022 and for 3 subperiods of interest: *before the Dobbs leak* (December 24, 2021-May 1, 2022), *Dobbs leak to decision* (May 2, 2022-June 23, 2022), and *after the Dobbs decision* (June 24, 2022-December 23, 2022). We used topic modeling to obtain descriptive topics for the year and each subperiod and then classified posts. Topics were then aggregated into *conceptual groups* based on a combination of quantitative and qualitative assessments. The proportion of posts classified in each conceptual group was used to assess change in community interests across the 3 study subperiods.

**Results:**

The 7273 posts shared in r/abortion in 2022 included in our analyses were categorized into 8 conceptual groups: abortion decision-making, navigating abortion access barriers, clinical abortion care, medication abortion processes, postabortion physical experiences, potential pregnancy, and self-managed abortion processes. Posts related to navigating access barriers were most common. The proportion of posts about abortion decision-making and self-management changed significantly across study periods (*P*=.006 and *P*<.001, respectively); abortion decision-making posts were more common before the *Dobbs* leak, whereas those related to self-management increased following the leak and decision.

**Conclusions:**

This analysis provides a holistic view of r/abortion posts in 2022, highlighting the important role of online communities as abortion-supportive online resources and changing interests among posters with abortion policy changes. As policies and pathways to abortion access continue to change across the United States, approaches leveraging natural language processing with sufficiently large samples of textual data present opportunities for timely monitoring, with the potential to reflect a broad range of abortion experiences, including those of people who have limited or no interaction with clinical abortion care.

## Introduction

### Overview

Abortion access in the United States has been in a state of rapid change and increasing restriction since the *Dobbs v Jackson Women’s Health Organization* (*Dobbs*) decision in June 2022 [1]. While many people across the United States faced various challenges to accessing abortion before *Dobbs*, with this decision, the legal protection of abortion at the federal level in the United States was removed, returning control to individual states [2]. Since this change, >20 states have entirely or largely banned legal abortion [3], abortion clinics have closed across the United States, remaining and emerging clinics have struggled with the burden of demand resulting in long wait times, and many people have been unable to access in-clinic abortions [4-6]. Given the relative recency of *Dobbs*, the progressive and rolling impacts that have resulted from that decision, and the potential for a national ban on abortion, researchers seek to monitor the impacts of *Dobbs* to provide timely insights [5,7,8]. Research using online information and leveraging methods to work efficiently with large datasets of user-generated text has the potential to accelerate knowledge generation and offer meaningful insights into online behaviors and changing interests related to abortion. We present background describing the importance of online health communities related to abortion and research exploring people’s use of these communities, with a specific interest in how natural language processing (NLP) techniques can further this area of research.

### Use of the Internet and Online Health Communities for Abortion

People experienced various challenges to abortion access before *Dobbs*, and as such, they sought support to help navigate the processes of abortion decision-making and access [9]. Many people discover pregnancies and make decisions about abortion outside of clinical contexts [10]. In addition, there are many people considering, seeking, and having abortions in the United States who never interact with formal clinical abortion care [11-13]. The internet, particularly with increasing accessibility in recent decades, plays an important role in people’s abortion decision-making and navigation. Research has shown that the internet is an important source of abortion information among adults in the United States, particularly for people living in contexts with more abortion restrictions [10,14-18]. With further constraints on abortion access, the internet is likely playing an increasingly important role in people’s abortion trajectories as a source of information, peer interaction, social support, health services, and medication [10,14-17]—but more information is needed about the specifics of its use since *Dobbs*.

Online health communities, or online groups of people with a common health interest or purpose governed by a set of policies or norms, are one internet-based resource that people use for abortion-related support [19]. Reddit is a popular social networking site used by approximately one-quarter of adults in the United States in 2022 [20]. Within Reddit, user-generated content is aggregated and dispersed across millions of user-created and monitored message boards on specific subjects (or subreddits; r/subject) to which users can subscribe as members. Members can post material (text, images, videos, or links) with generous character count limits (40,000 characters, approximately 5000-10,000 words), which other members interact with using comments and an upvote and downvote system. Each subreddit is governed by a set of moderators elected from within the community who set subreddit-specific guidelines for posting content, review posts to ensure that those guidelines are being met, and have the option to delete noncompliant posts and ban deviant users. Some subreddits have developed around health topics and function as online health communities.

We chose Reddit as the focus for this research given its popularity and the uniquely valuable window that it offers into how people discuss health concerns outside of formal research settings or clinical encounters. Reddit provides anonymity with its use of pseudonyms and usernames, lack of requirement for users to share personal information in the creation of profiles, lack of collection of IP address information, and lack of restriction on the use of “throwaway” accounts (ie, temporary accounts created for a specific, single use or short-term purpose). These features allow users to engage in more open and honest discussions than they might on other social networking sites [21-23] or with family members or health care providers [24-26]. As a public, user-driven platform, Reddit enables peer-to-peer information exchange and emotional support, often among individuals who may face stigma or lack access to trusted clinical support. This makes it particularly appealing for communication about stigmatized and politicized health experiences such as abortion.

The r/abortion subreddit is a community that aims to “offer support and advice to people who are seeking or have had an abortion.” In 2022, r/abortion had almost 45,000 members and was actively moderated by the Online Abortion Resource Squad (OARS), a group that trains volunteers and works to offer consistent, quality information and support to people coming to r/abortion [27]. The group of moderators working with OARS also actively enforces the r/abortion community rules. Previous research has established that people use online communities, particularly those on Reddit, to communicate about abortion decision-making; challenges to abortion access; taking action to end a pregnancy outside of clinical contexts, or self-managed abortion (SMA); sharing experiences and advice; and needs for support [28-37]. These studies have used qualitative methods to analyze a defined subsample of content, with either a topical query (eg, mention of “abortion” [33]) or a narrow temporal focus (eg, posts from the previous 2 weeks [28,34]). While these qualitative analyses have allowed researchers to explore the nuances of abortion experience and communication in online communities, their scopes provide limited information about the range of uses for this community or any changes in use over time.

### Abortion Research Using Machine Learning and NLP Techniques

Researchers with an interest in health behaviors and outcomes have quantitatively explored the use of online communities, including subreddits, for some topics related to sexual and reproductive health, including patient perceptions of prenatal diagnostic testing [38], disclosure and use of throwaway accounts when posting about parenting [39], perceptions of the human papillomavirus vaccine [40], discussion of sexually transmitted diseases [41], the use of message boards related to miscarriage and stillbirths [42], and discussions of contraception [43]. Some of these analyses have used machine learning tools to analyze the textual data. Similarly, other projects have used computational methods to explore discussions of abortion on social networking sites with an interest in assessing and improving research approaches, often using abortion as a test case for a polarizing and complex topic of communication in these spaces [44-47]. In addition, a recent study by Valdez et al [48] explored a subset of posts to r/abortion and r/AbortionDebate in early 2022 and found that these online communities were used differently, with r/abortion often serving as a place to seek and share abortion support and r/AbortionDebate as a platform to debate abortion attitudes. These quantitative analyses and the qualitative approaches described previously demonstrate that data from online communities can provide insights into population interests and needs, as well as into how these communities serve as sexual and reproductive health resources.

However, research using machine learning tools with an explicit interest in abortion as a health concern impacted by sociopolitical context is lacking. Machine learning tools present a particularly compelling opportunity to advance public health research, providing timely and holistic information about how people have used online communities for abortion. Machine learning has a range of applications, including speech recognition, medical diagnosis, and NLP [49]. NLP seeks to process and analyze large amounts of language data—both text and spoken—to understand, interpret, and generate human language meaningfully and with consideration of context. There are many techniques within NLP, including tokenizing text into smaller units, recognition and tagging of parts of speech, text classification, topic modeling, and word embeddings [49,50]. Similar to other machine learning techniques, NLP can be carried out with varying levels of human input. However, when human expertise is particularly crucial, a “human-in-the-loop approach”—or a collaborative approach in which humans and machines work together—can be used [51]. NLP techniques create the potential for accelerated analyses of large samples of language data compared to traditional qualitative analyses and have been leveraged by public health researchers to explore a range of topics [38,39,41,43,45,52-56]. However, to date, there are no applications of NLP techniques to analyze online communication about abortion focused on the *Dobbs* decision as a public health concern, leveraging content expertise with a human-in-the-loop approach.

### Research Aims

With ongoing changes to abortion access in the United States and people’s use of online communities for abortion, research exploring how people use these communities can provide important insights for researchers, care providers, advocates, and policy makers. Past research has explored relatively narrow aspects of online community use related to abortion or as a topic for a research case study. However, there is a need for a broader understanding of how this type of online community is used for abortion and how it may reflect changes in the sociopolitical and legal context of abortion access. Particularly given the rapidly changing landscape of abortion policy and access in 2022, with uncertainty and increasingly constrained pathways to care, online resources likely play a key role in providing access to information, support, and services. In addition, exploring people’s use of online communities for abortion has the potential to provide timely insights from populations that may be hard to reach and not well represented by much of the research on abortion experiences [57-59].

Using NLP techniques to analyze text shared in online abortion communities can help address these gaps, allowing for efficient analysis and pattern detection in the large bodies of user-generated narratives shared in these digital spaces. As such, this research sought to use NLP techniques to explore the content of posts to 1 online community for abortion (r/abortion) in 2022 and assess whether the use of the community changed during that time. Specifically, we used unsupervised topic modeling on posts submitted during the year surrounding the *Dobbs* decision (6 months before and after June 24, 2022) to inductively discover the topical content of posts, integrating a human-in-the-loop approach in the quality review, naming, and aggregation of topics.

## Methods

### Overview

Reddit data have historically been accessible through free, publicly available application programming interfaces (APIs). Researchers have often used Pushshift’s Reddit API and Reddit’s official API to obtain compiled information about content shared on Reddit, including creation date, submission (post or comment) text, community interactions (likes, upvotes, and downvotes), and more. Each API had different capabilities but generally provided access to organized and cataloged Reddit data that could be queried to obtain datasets for analysis.

A previous analysis of r/birthcontrol used data obtained from Pushshift’s Reddit API [60], as have various other analyses focused on health-related use of Reddit [54-56,61-63]. Given changes to Pushshift’s Reddit API in December 2022, specifically issues preventing direct access to any post submission data from before November 3, 2022, we used 2 different data access approaches to obtain data for this analysis—one for data from December 24, 2021, to August 23, 2022 (gathered and stored for cleaning and analysis in 2022), and another for data from August 23, 2022, to December 23, 2022 (gathered and stored for cleaning and analysis in 2023). Notably, in April 2023, Reddit changed its data access policies, so public access to complete Reddit data through APIs, and the methodology outlined in this manuscript, is obsolete. We chose a start date of December 24, 2021, for this analysis to provide data for 6 months before and 6 months following the Dobbs decision. Details of the data collection approach and methods used for this analysis are presented in Multimedia Appendix 1 [64-68].

**Figure 1:**
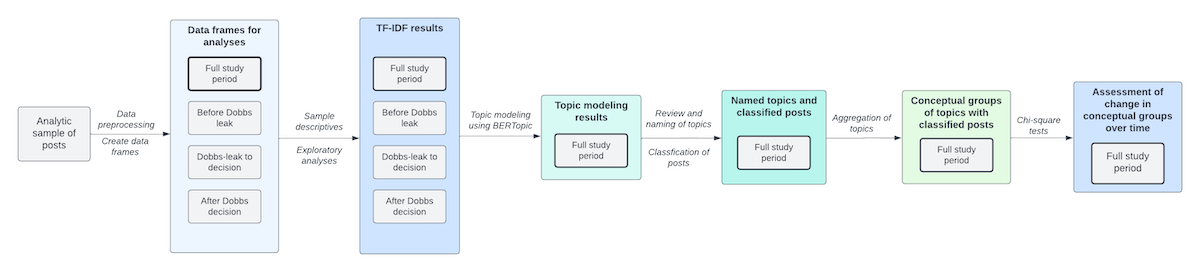
Methods process flow for natural language processing of posts to r/abortion from December 24, 2021, to December 23, 2022. TF-IDF: term frequency–inverse document frequency.

A visual overview of the analytic approach used in this research is presented in Figure 1. After procuring complete data from Reddit’s API, we implemented additional restrictions to obtain the analytic sample used for this research. These restrictions aimed to provide a sample of posts theoretically within the public domain, containing sufficient text to support contextualized NLP analysis. Sequentially, we excluded posts if they were removed, were deleted, contained only an image, contained only a link, or contained <30 characters. We then cleaned content in this analytic sample to remove usernames, which were replaced with a unique submission ID created for this analysis. These deidentified data were used for the analyses described. The broad sample of posts obtained provides a holistic view of the topics discussed by r/abortion community members in relation to their abortion-related questions, experiences, challenges, and more following the Dobbs decision and related changes in abortion access in the United States in 2022. Comments responding to original posts were not included in this analysis.

To prepare our data for NLP analyses, we cleaned data from posts using part-of-speech tagging, removing links, removing punctuation, changing all text to lower case, removing words with ≤2 characters, and removing stop words.

### Analysis

We began this analysis by counting all included posts submitted to r/abortion during the study period by date to summarize the community’s overall use during the year. We then described the textual data from posts in our analytic sample for the year-long study period using the following: count of posts (by day and study period), average word count of text in posts, number of unique accounts that posts were made from, and average number of posts per author.

We determined distinctive words and phrases in posts using count vectorization [64,65], applying word frequency analysis to posts from the year and within 3 subperiods of interest in 2022 for this research: *before the Dobbs leak* (December 24, 2021-May 1, 2022), Dobbs leak to decision (period from the *Dobbs* leak to the decision; May 2, 2022-June 23, 2022), and *after the Dobbs decision* (June 24, 2022-December 23, 2022).

### Topic Modeling

#### Overview

Topic modeling can discover latent topics, or themes, in documents. For this analysis, each post was defined as a document for topic modeling, and each data frame represented a set of documents. BERTopic was used given its capacity to account for the contexts of words in sentences in text, extending traditional topic modeling approaches that do not account for the semantic relationships between words [66]. It also provides various opportunities to explore topic hierarchies, topic visualization, and topic analyses. BERTopic was applied to preprocessed post text. Topic numbers, labels (top 10 words), and associated documents were reviewed by a research assistant and the lead researcher to assess model coherence. We manually reviewed a random sample of approximately 20% of all posts for a topic unless a topic had <50 posts, in which case review was completed for all posts in that topic. During this review process, we also developed and assigned a descriptive name for each topic based on the topic label and the raw text of representative submissions. Outlier documents were grouped into an outlier topic group (labeled as −1) in the modeling process and excluded from further review and analysis [67].

While BERTopic generates topics under the assumption that documents can include multiple topics, our categorization approach only assigned each submission to the topic that it had the highest probability of being in (rank 1 topic classification). This approach provided a single topic assignment for each document, reflecting the single most dominant topic describing that post. We reviewed posts classified under each topic to check the quality of assignments and then obtained counts to ascertain the number of posts classified under each topic. Using the trained models, we categorized all r/abortion posts using predictions in BERTopic [68]; classification was conducted using raw post text.

Topic modeling was carried out inductively, yielding results that we reviewed to determine a rigorous and consistent approach to topic aggregation. After reviewing topic labels and representative documents, it was clear that, while many topics used distinctive words, they described similar concepts. As such, we were interested in aggregating topics into *conceptual groups*, or clusters of related topics grouped into broader themes or domains that described key concepts represented by the topics in each model. Conceptual group assignments were manually determined for all topics using an approach that combined dendrogram results (quantitative) with a manual review of submission texts (qualitative), with a focus on defining conceptually meaningful and interpretable groups of topics. Post counts for each conceptual group were obtained based on the summed rank 1 classification assignments for topic modeling results, summarizing the commonality of submissions in each conceptual group.

#### Assessing Changes in Conceptual Group Frequency Over Time

We used counts of posts in each conceptual group for the year to assess the proportion in each group and differences between the subperiods of interest (*before the Dobbs leak*, *Dobbs leak to decision*, and *after the Dobbs decision*). We assessed statistically significant differences in the proportion of posts in a conceptual group using chi-square tests in R (*chisq.test* package; R Foundation for Statistical Computing). This compared the proportion of posts in a conceptual group versus those not in that conceptual group across the 3 study subperiods, assessing whether there was a change in the frequency of posts primarily focused on that concept throughout 2022. This component of the analysis sought to describe any changes in the primary focus of r/abortion submissions during 2022 considering the dramatic changes to the legal and social environment for abortion during the year

### Ethical Considerations

The collection and analysis of these data was exempted from review by the Committee for Protection of Human Subjects at the University of California, Berkeley (2022-08-15585). As data were publicly available and Reddit content is accessible without an account, we did not obtain informed consent from r/abortion users. However, users may still engage with this platform with the expectation of privacy, and ethical principles related to informed consent, participant confidentiality, and privacy still arise when using data from Reddit [69-71]. These are of particular concern given the sensitivity of abortion narratives and the potential for digital data to be used to support abortion-related prosecution [72,73]. While past research using Reddit data has used techniques such as the exclusion of usernames and rephrasing or paraphrasing of post text to protect user identities [53,74,75], these approaches have generally been found to be insufficient to protect user identities, with the capabilities of various online search tools to discover users through the content they posted [76]. The current best practice when conducting research using Reddit data is to engage in a “heavy disguise” process with rigorous testing to produce disguised stories, as described by Reagle [76]. These concerns are especially pressing in the post-*Dobbs* era, where digital information may be weaponized in legal proceedings.

To mitigate these risks, we adhered to the current best practices for ethical internet research using sensitive data on health topics. We maintained the name of the subreddit that the data were obtained from given its visibility as an abortion community on Reddit and its relatively high number of members, offering some level of shielding for individual users [69]. While all analyses were carried out using complete text from submissions, we used an ethical fabrication process to develop representative narratives called “composite quotes” [76,77]. We gathered representative posts for a specific topical area of our findings and used them to generate a composite narrative related to that particular facet of an abortion experience. We replaced keywords, adjusted sentence ordering, and combined details of experiences across individuals. Once a composite quote was generated, its discoverability was checked by searching the overall quote and each sentence on Google (including the terms “Reddit AND abortion AND [the searched text]”), Reddit, and the Pushshift Reddit Search Tool [76,78]. Once composite quotes were checked via searches, a plagiarism checker was used as a final check to ensure that these stories were effectively nondiscoverable using current tools [79]. As such, all stories presented throughout this paper are composite quotes intended to reflect and represent the narratives shared in r/abortion but include a degree of ethical abstraction. This approach reflects a broader ethical stance that recognizes the public and private ambiguity of online data and the evolving legal risks of sharing abortion experiences online. Research in this area must not only comply with institutional review board standards but also grapple with new forms of digital vulnerability, especially in politically charged contexts.

An interdisciplinary team of researchers and advocates conducted this research, grounded in the principles of reproductive justice and the premise that abortion access is a health and human rights concern. To ensure transparency and provide context for our perspectives, we offer individual positionality statements. EP is a female-identifying individual who resided in an abortion-protective US state during the research. She is a Reddit user who primarily reads content. NP is a female-identifying individual who lived in an abortion-protective US state at the time of the study. She does not use Reddit but brings extensive experience working on sexual and reproductive health in abortion-restrictive countries. CM is a female-identifying person who lived in an abortion-protective US state at the time of this research; she is not a Reddit user. UU is a female-identifying person who lived in an abortion-protective US state at the time of this research; she is a Reddit user who primarily reads content. CC is a male-identifying individual who resided in an abortion-protective US state during the research period. He is an active Reddit user.

Our team brought varying levels of experience with Reddit, which enriched our engagement with data from r/abortion and informed our analysis. While all team members were living in abortion-protective states during the study, several of us have personal or professional ties to regions with more restrictive abortion policies. These experiences influenced how we approached the data, interpreted the results, and framed our findings.

## Results

### Descriptive Statistics

Of the 12,509 posts shared in r/abortion during the study period (December 24, 2021-December 23, 2022), 2351 (18.79%) were deleted by the users, 1349 (10.78%) were deleted by the user and removed, and 1484 (11.86%) were removed (Table 1). Therefore, 41.44% (5184/12,509) of all posts were elided content and not eligible for analysis. Of the eligible posts, only 0.01% (1/12,509) contained only a link, and a small portion (51/12,509, 0.4%) contained <30 characters. This provided an analytic sample of 7273 eligible posts for the study year.

**Table 1:** Analytic samples of posts for the year and each subperiod, with counts of elided and excluded submissions (N=12,509).

			Before the *Dobbs* leak (December 24, 2021-May 1, 2022; n=3650)		*Dobbs* leak to decision (May 2, 2022-June 23, 2022; n=1828)		After the *Dobbs* decision (June 24, 2022-December 23, 2022; n=7031)		Year total
**Elided content^a^, n (%)**			1586 (43.45)		829 (45.35)		2769 (39.38)		5184 (41.44)
	Deleted by user^b^	572 (15.67)		267 (14.61)		1512 (21.5)		2351 (18.79)	
	Deleted by user+removed^b^	867 (23.75)		288 (15.75)		194 (2.76)		1349 (10.78)	
	Removed^b^	147 (4.03)		274 (14.99)		1063 (15.12)		1484 (11.86)	
**Posts excluded from the sample, n (%)**			11 (0.3)		4 (0.22)		37 (0.53)		52 (0.42)
	Contained only an image	0 (0)		0 (0)		0 (0)		0 (0)	
	Contained only a link	1 (0.03)		0 (0)		0 (0)		1 (0.01)	
	<30 characters	10 (0.27)		4 (0.22)		37 (0.53)		51 (0.41)	
Elided+excluded posts, n (%)			1597 (43.75)		833 (45.57)		2806 (39.91)		5236 (41.9)
Posts in sample, n (%)			2053 (56.25)		995 (54.43)		4225 (60.09)		7273 (58.14)
Posts per day, median (IQR)			16.0 (5.5)		18.0 (5.0)		23.0 (8.0)		19.0 (8.0)

^a^The proportion of content removed by Reddit in this case aligned with previous accounts from Reddit on their removal of content (6% in 2020). While there is no systematic accounting of content deleted from Reddit, research on the 3 most popular sensitive-topic subreddits found that approximately half of the content was deleted by users [80], a higher proportion than that observed in these data from r/abortion.

^b^Deleted content refers to posts removed from Reddit by the original poster. Removed refers to posts that were removed by Reddit or r/abortion moderators, and Deleted by user+removed refers to content that was both removed by Reddit or r/abortion moderators and then deleted by the user. Categories are exclusive.

**Figure 2:**
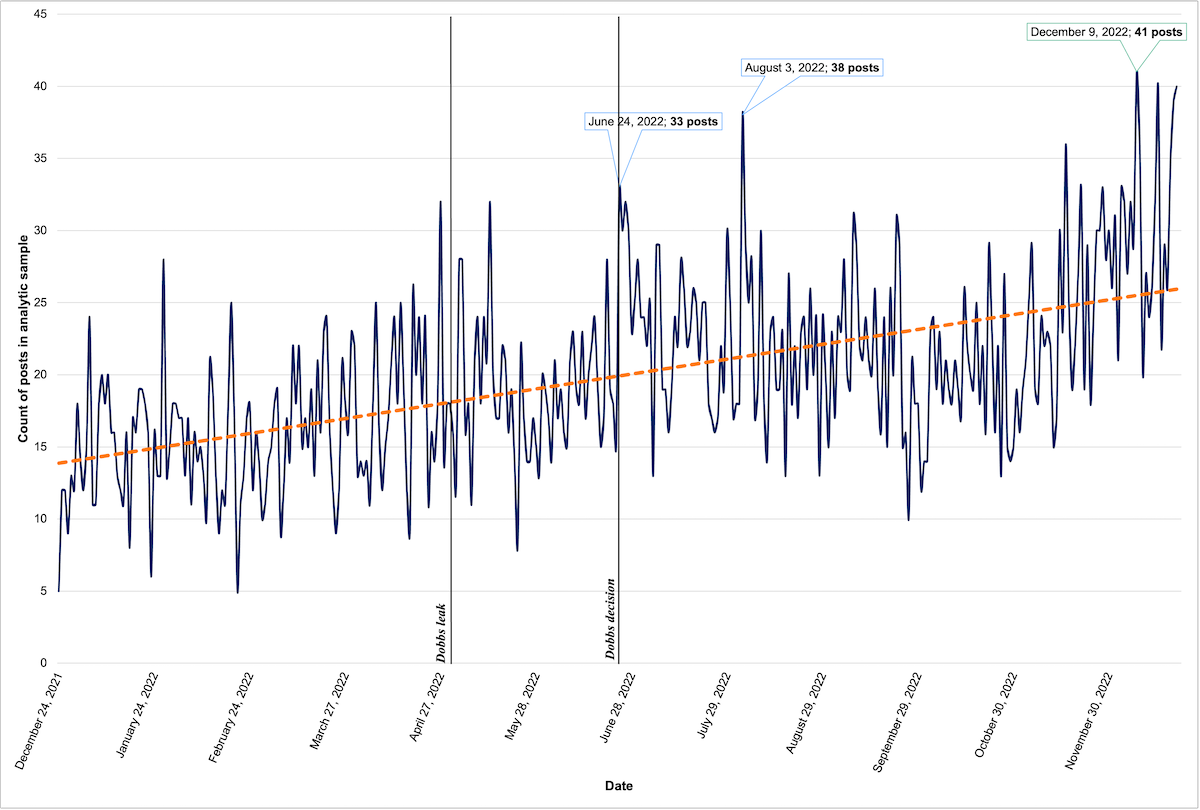
Line graph showing daily volume of posts to r/abortion in 2022, with markers for the dates of the Dobbs leak and decision (N=7273 posts). The highest daily volume of posts was observed on December 9, 2022 (n=41 posts). Other peak dates for post submissions were June 24, 2022 (n=33 posts; the date of the Dobbs decision), and August 3, 2022 (n=38 posts).

The highest daily volume of posts was on December 9, 2022, with 41 eligible submissions (Figure 2). As there were no substantive policy, political, or social events related to abortion at this time (except for the legalization of abortion in Argentina), we believe that this peak is a random date in a larger upward trend. Other peak dates for post submissions were June 24, 2022 (33 posts; the date of the *Dobbs* decision), and August 3, 2022 (38 posts). The median number of eligible posts throughout the year was 19 (range: 5-41, IQR 8) per day, and the median number of posts per period increased throughout the year, with 16 posts per day before the *Dobbs* leak, 18 posts per day between the leak and the decision, and 23 posts per day following the *Dobbs* decision. Posts in the analytic sample had a median of 53 words per submission across the year (range 2-3204).

Posts were shared from 4468 unique accounts during the year, with posts made by 1379 users before the leak, 702 users between the leak and the *Dobbs* decision, and 2620 users following the *Dobbs* decision. On average, posters shared <2 posts in the r/abortion community during the year from a single Reddit user account (mean 1.6; IQR 0.5-1.6), with slight variation across subperiods.

### Analyses of Cleaned Submission Text

#### Exploratory Data Analyses: Count Vectorizer Results

We obtained distinguishing sequences of consecutive words of different lengths, known as uni-, bi-, and trigrams (“n-grams”), from posts. These topics included abortion decision-making timing and feelings, which commonly included “feel,” “want,” “baby,” “know,” “pregnant,” and other words (see Multimedia Appendix 2) [81] for the year and by study period in relation to the *Dobbs* leak and decision. For the year overall (N=7273 posts), n-grams reflect discussions of pregnancy and pregnancy confirmation, abortion timing and decision-making, and access through clinic-based and online care. Throughout 2022—in yearly results and across subperiods—n-grams reflect discussions of pregnancy and abortion decision-making and experiences during abortion processes. These n-grams also highlight consideration of changes related to *Dobbs* and the overturning of *Roe v Wade*, particularly access while living in a restrictive setting, with the emergence of related language starting from *Dobbs* leak.

#### Topic Modeling

##### Overview

Posts from the yearly sample (N=7273) were described through 55 inductively generated topics with 3 outlier topics (in the −1 outlier group, see Table 2). These topics contained between 10 and 472 words, reflecting a range of word density across topics. On the basis of a review of topic labels and representative documents, these topics reflect that the analyzed posts described a wide range of abortion-related experiences and concerns—summarized through topic names. These topics included *abortion decision-making timing and feelings*, which commonly included “feel,” “want,” “baby,” “know,” “pregnant,” and other words. *Appointments and cost barriers* was another topic describing yearly posts, including “abortion,” “help,” “get,” “get abortion,” and “appointment” as common words. In addition, people posted about *medication abortion, physical process bleeding, and clots* using words such as “bleeding,” “clots,” “cramps,” “took,” “passed,” and “blood.” There were also topics such as *Aid Access shipping*, where posters used words such as “package,” “customs,” “tracking,” “usps,” and “aid access.”

##### Document Classification Using Topics and Aggregation Into Conceptual Groups

Topics describing posts from the study year (n=55) were used to classify all posts (N=7273) into their highest-probability topic. The count and proportion of posts classified under each topic are presented in Table 2. Topics with the highest number of classified posts were *seeking access to medication abortion* (2408/7273, 33.11% of posts); *abortion decision-making, sharing stories, and seeking support* (657/7273, 9.03% of posts); and *medication abortion process, timing of pills* (328/7273, 4.51% of posts).

We aggregated these topics into 8 conceptual groups: *abortion decision-making*, *navigating access barriers*, *clinical abortion care*, *medication abortion process*, *postabortion physical experiences*, *potential pregnancy*, and *SMA process*. On the basis of conceptual group classification counts (summed topic classification counts), *navigating access barriers* was the conceptual group with the largest number of posts (2446/7273, 33.63% of all posts), which included posts describing various experiences with challenges seeking and having an abortion. Posts focused on aspects of the *medication abortion process* were the second most common (1807/7273, 24.85%), followed by *abortion decision-making* (974/7273, 13.39%), *postabortion physical experiences* (672/7273, 9.24%), *clinical abortion care* (670/7273, 9.21%), *potential pregnancy* (550/7273, 7.56%), and *SMA process* (151/7273, 2.08%).

Composite versions of representative posts for each conceptual group are presented in Multimedia Appendix 2. Select quotes for each conceptual group are presented in this section, representing the content of posts in each group for the year overall and the comparable subperiods. Some posts in the *navigating access barriers* conceptual group described difficulty obtaining an appointment at an abortion clinic, sharing stories such as the following:

My boyfriend and I live in the south with our 9-month-old baby, and we just found out I’m pregnant again. We really can’t afford to and aren’t prepared to have another child. Abortion is illegal in our state and we live 4 hours from the nearest clinic in the nearest legal state. Do we have any other options?

Composite quote

Others focused on challenges in accessing medication abortion, often describing specific barriers and sometimes asking for information and resources to help them access it, as with posts such as the following:

My girlfriend and I are in urgent need of abortion pills. She’s about 3 or 4 weeks pregnant and we want to get through this asap and for not a lot of money, preferably for a few hundred dollars or less. Can anyone provide some info and walk us through the process?

Composite quote

Others specifically referenced concerns or issues with abortion access related to *Roe v Wade* and the *Dobbs* decision, with narratives such as the following:

I think I might be pregnant and I’m really scared, especially after hearing Roe v Wade might be overturned. I’m thinking of having an abortion but I live in a state where abortion will likely become illegal right away. Does anyone know if I will still be able to get an abortion somehow? I’ve been trying to research online and have been seen a lot of different information. Are there any online clinics? Would it be illegal to travel to another state to go to a clinic in-person? I’ve heard that Colorada or Illinois might be safe options but I’m just really terrified. I just want to be sure I can access an abortion no matter what happens.

Composite quote

Among posts describing the *medication abortion process,* there were many asking questions about or descriptions of the use of pills to have a medication abortion. These posts sometimes included specific questions about correct timing in the use of medication abortion, sharing stories such as the following:

I ordered pills through AidAccess and they arrived today. I’ve seen online that you’re supposed to wait 24 hours after you take the mifepristone to then take the misoprostol, but on the box it doesn’t mention timing and the instructions are very vague. I don’t want to screw this up at all so can someone clarify the timing and what exactly I should do?

Composite quote

People also commonly posted about *abortion decision-making.* In these posts, people often described their pregnancy experiences and abortion story while seeking community support, with narratives such as the following:

Can anyone share about their experiences during and after their abortions around 6 weeks? I’m trying to pick between medication or surgical. My last abortion was surgical and it was not a great experience. I just want this to be over with because ideally, I wouldn’t want to have to go through this, but I am not in a place to support a child financially.

Composite quote

Posters also wrote about making their abortion decision in relation to their relationship dynamics, with narratives such as the following:

I can’t take this anymore, I’m going to get an abortion. I’m just not ready to have a baby and I know that this will be for the best. I’m not looking forward to having to face my partner after this. I’m afraid he’s going to hate me for having an abortion and break up with me.

Composite quote

Posts asking questions about and describing *postabortion physical experiences* were also common, with posters describing experiences with bleeding or clots after their abortion and often seeking input from the community on how normal their experiences were:

I took my miso last night and have been bleeding for almost 12 hours now. I had cramping for the first few hours then a big flow of blood where I passed two big clots the size of small lemons. But those are the only clots I’ve had and I haven’t been bleeding any more. I know you’re supposed to pass a lot of clots based on what I’ve been reading. Should I be worried?

Composite quote

Posts also described aspects of *clinical abortion care*, including specific facets of clinical care—particularly ultrasound—and fears and concerns about having an abortion. Posts also focused on *potential pregnancy*, sometimes describing sexual experience and possible risk of pregnancy. In contrast, others focused on potential pregnancy after an abortion in relation to still testing positive weeks later. In addition, some posts described *experiences with SMA*, often focused on the process of ordering medication and having it shipped while asking for support from the community in navigating those processes.

**Table 2:** BERTopic topic modeling classifications and conceptual group aggregations for yearly posts with topic names, rank 1 classification counts, conceptual groups, and summed rank 1 conceptual group classification counts (N=7270 posts; 3 posts excluded in the −1 outlier group).

Conceptual group and topic name		Topic posts, n (%)^a,b^	Conceptual group posts, n (%)^c^	
**Navigating access barriers**				2446 (33.7)
	“Appointments and cost barriers”	4 (0.2)^d^		
	“Seeking access to medication abortion”	2408 (98.5)^d^		
	“Dobbs decision barriers”	21 (0.9)^d^		
	“Appointments and travel barriers”	13 (0.5)^d^		
**Medication abortion process**				1807 (24.9)
	“Medication abortion process, timing of pills”	328 (18.2)^e^		
	“Medication abortion normal physical process”	122 (6.8)^e^		
	“Medication abortion physical process misoprostol”	299 (16.6)^e^		
	“Medication abortion physical process miso”	51 (2.8)^e^		
	“Medication abortion process pill administration”	10 (0.6)^e^		
	“Medication abortion process timing”	7 (0.4)^e^		
	“Medication abortion process taking pills”	276 (15.3)^e^		
	“Medication abortion physical process bleeding and clots”	31 (1.7)^e^		
	“Medication abortion physical process mifepristone and misoprostol”	90 (5)^e^		
	“Medication abortion physical process and completion”	205 (11.3)^e^		
	“Medication abortion process, seeking information about access and use experience”	304 (16.8)^e^		
	“Medication abortion physical process pain and clots”	7 (0.4)^e^		
	“Nausea during pregnancy and abortion”	77 (4.3)^e^		
	“Abortion decision-making postabortion grief and regret”	31 (1.7)^e^		
	“Abortion decision-making postabortion timing and regret”	3 (0.2)^e^		
	“Abortion decision-making, sharing stories and seeking support”	657 (36.4)^e^		
	“Abortion decision-making support (presence and lack)”	70 (3.9)^e^		
	“Abortion decision-making, reflection and navigating challenges”	49 (2.7)^e^		
	“Abortion decision-making and pregnancy confirmation”	4 (0.2)^e^		
	“Abortion decision-making fear and support”	2 (0.1)^e^		
	“Abortion decision-making relationship dynamics and possible pregnancy”	36 (2)^e^		
	“Abortion decision-making relationship dynamics”	122 (6.8)^e^		
**Postabortion physical experiences**				672 (9.2)
	“Post medication abortion bleeding and menstruation”	89 (13.2)^f^		
	“Post abortion bleeding”	58 (8.6)^f^		
	“Post abortion bleeding and menstruation”	44 (6.5)^f^		
	“Post abortion bleeding and clots”	50 (7.4)^f^		
	“Post procedural abortion physical experiences”	29 (4.3)^f^		
	“Post abortion breast changes”	51 (7.6)^f^		
	“Post abortion menstruation”	35 (5.2)^f^		
	“Post procedural abortion bleeding”	123 (18.3)^f^		
	“Post abortion clots”	193 (28.7)^f^		
**Clinical abortion care**				670 (9.3)
	“Medication abortion clinical care”	1 (0.1)^g^		
	“Procedural abortion fears”	59 (8.8)^g^		
	“Accessing clinical abortion care”	72 (10.7)^g^		
	“Clinic protestors”	4 (0.6)^g^		
	“Clinical abortion care recovery”	15 (2.2)^g^		
	“Abortion completion and ultrasound”	39 (5.8)^g^		
	“Abortion experience fears”	244 (36.4)^g^		
	“Abortion and ultrasound”	236 (35.2)^g^		
**Potential pregnancy**				550 (7.6)
	“Potential pregnancy testing”	226 (41.1)^h^		
	“Abortion completion pregnancy testing”	87 (15.8)^h^		
	“Pregnancy risk and sex”	144 (26.2)^h^		
	“Pregnancy risk and advice”	2 (0.4)^h^		
	“Potential confirmation negative tests”	91 (16.5)^h^		
**SMA** ^i^ **process**				151 (2.1)
	“SMA in illegal settings”	5 (3.3)^j^		
	“Aid Access shipping”	50 (33.1)^j^		
	“Aid Access ordering”	43 (28.5)^j^		
	“Aid Access ordering and credibility”	11 (7.3)^j^		
	“Aid Access ordering and shipping”	6 (4)^j^		
	“SMA online navigation”	22 (14.6)^j^		
	“SMA outside of the US”	14 (9.3)^j^		

^a^*Topic posts* refers to the count of posts classified under this topic based on rank 1 (highest-probability) classification.

^b^In total, 3 topics generated had 0 posts classified under them based on rank 1 (highest probability) and were dropped from the results. These topics were abortion decision-making timing and feelings, abortion decision-making desired children, and pregnancy risk and menstruation.

^c”^Conceptual group posts” refers to the count of all posts in this conceptual group. The percentages presented indicate the proportion of all posts in the sample in this conceptual group based on topic classification and conceptual group aggregation.

^d^n=2446.

^e^n=1807.

^f^n=672.

^g^n=670.

^h^n=550.

^i^SMA: self-managed abortion, defined for this research as taking action to end a pregnancy outside of the formal health care system with or without clinical support, which includes the use of safe medications such as misoprostol and mifepristone but also potentially harmful or ineffective methods [81].

^j^n=151.

##### Assessing Changes in Conceptual Group Frequency Over Time

**Figure 3:**
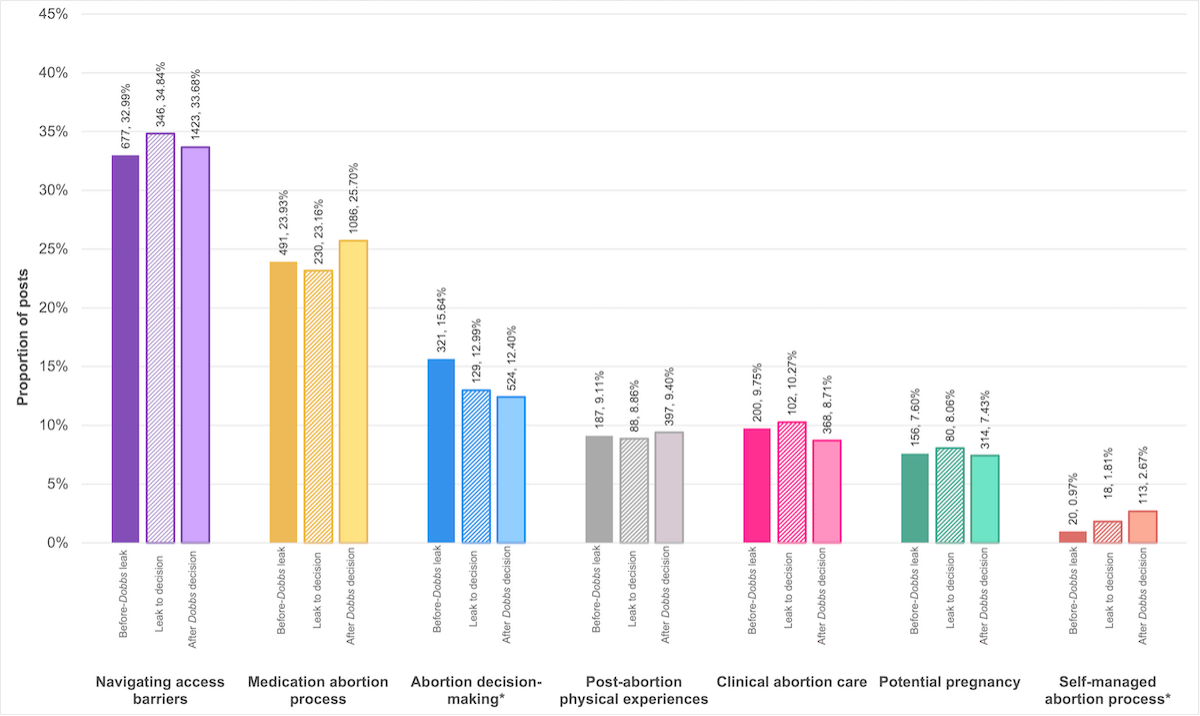
Bar graph showing the proportion of r/abortion posts in each conceptual group (7 in total) in each study subperiod—before the Dobbs leak, Dobbs leak to decision, and after the Dobbs leak. The largest proportion of posts was in the navigating access barriers conceptual group, followed by medication abortion process and abortion decision-making. The proportion of posts about abortion decision-making changed significantly across subperiods, decreasing over time. Posts about self-managed abortion were the least common, but the proportion also changed across subperiods—increasing over time (N=7270 posts). *Indicates that the proportion of posts for this conceptual group varied significantly across study subperiods based on a Pearson chi-square test.

Counts and proportions of posts in each conceptual group across study subperiods based on the highest-probability (rank 1) classification, along with comparisons of proportions, are presented in Figure 3, with additional information in Multimedia Appendix 2. Overall, the number of posts to r/abortion increased after *Dobbs*. The proportion of posts related to *abortion decision-making* changed significantly across study periods (*P*=.002); with 974 posts in this group, 33% were from before the *Dobbs* leak (n=321); 13% from *Dobbs* leak to decision (n=129); and 54% after the *Dobbs* decision (n=524). In addition, while posts related to SMA were the least common, the proportion of posts in this group also changed significantly across study subperiods *(P*<.001), with 151 posts in this group, 13% were from before the *Dobbs* leak (n=20), 12% from *Dobbs* leak to decision (n=18), and 75% after the *Dobbs* decision (n=113). Changes across the study subperiods were not significant for other conceptual groups: *navigating access barriers* (*P*=.60), *medication abortion process* (*P*=.13), *post-abortion physical experiences* (*P*=.85), *clinical abortion care* (*P*=.19), *potential pregnancy* (*P*=.80).

## Discussion

### Principal Findings

Given the rapidly changing landscape of abortion policy and access in 2022, with uncertainty and increasingly constrained pathways to care, online resources play a key role in providing access to abortion information, support, and services. Online communities in particular provide unique support for members of the public, as well as opportunities for researchers to understand emerging concerns and experiences of populations that may be hard to study through other methods [57-59]. We sought to contribute to the body of research that uses online information and leverages methods to work efficiently with large datasets to accelerate knowledge generation and provide novel insights into changing abortion-related experiences surrounding the *Dobbs* decision in 2022.

People posted on r/abortion about many different abortion-related concerns in 2022, coming to the community at various stages in their abortion trajectories. This ranged from posts seeking community input on pregnancy confirmation, abortion decision-making, pathways to access abortion, assessing abortion completion and postabortion experiences (physical and emotional), and support during and after their abortion in managing the process. Overall, the volume of posts and the breadth of content shared in them reflect that r/abortion is a community used abundantly and in alignment with its stated mission: “If you’re pregnant and don’t want to be, we can help you get an abortion. This is a proabortion, stigma-free space to ask questions, get information, and share your experiences” [82]. Given the increases in the use of r/abortion throughout 2022, this pseudonymous community may be an increasingly important online resource as abortion access continues to be restricted after *Dobbs.* Notably, other subreddits have substantially larger volumes of community members and posts; for example, r/TwoXChromosomes had >13 million members and between 80 and 450 posts per day in 2022 compared to r/abortion with 34,000 members and between 5 and 41 posts per day [83,84]. However, r/abortion is an active and uniquely abortion-supportive community on Reddit facilitated by an active network of moderators (with the OARS), playing an important role in the landscape of abortion resources.

Overall, we observed that the conceptual group with the highest volume of posts focused on navigating barriers to abortion access, and the proportion of posts in that group did not change significantly across subperiods in 2022. This illustrates that r/abortion posters described barriers to abortion access even before *Dobbs* introduced national challenges to the legality of abortion. Previous research extensively documents pre-*Dobbs* barriers to abortion access and their adverse impacts on timely abortion access and the health of birthing people and their children; our findings illustrate the sustained influence of barriers on the experiences of r/abortion posters [10,28,29,85-101]. Posts describing navigating barriers to abortion access largely described challenges in accessing medication abortion based on topic modeling results, with some discussing the *Dobbs* decision, appointments and travel barriers, and costs. The overwhelming focus on challenges in accessing medication abortion in our sample may reflect broader trends, with use of medication abortion increasing since it became available in the United States in 2001 and now accounting for >60% of all abortions [102]. It may also reflect the unique priorities of the population using r/abortion and the potential for online resources to facilitate direct access to medication abortion. These findings are echoed and expanded by a qualitative analysis by our team using a subset of data from r/abortion following the *Dobbs* leak, which found that people described a variety of structural and social barriers to abortion access, including emerging challenges such as concerns about legal risks associated with accessing abortion. These findings also highlight the negative impacts of barriers on timely access to the desired modality of abortion care and mental health, as well as self-management of abortion out of necessity [36].

We also found that the proportion of posts related to SMA and abortion decision-making changed significantly throughout 2022. Abortion decision-making posts were more common before the *Dobbs* leak, whereas those related to self-management increased following the leak and decision. The relative decrease in posts related to decision-making may reflect shifting interests in the community when faced with increasing challenges. The increasing interest in SMA in our sample is notable, highlighting increasing interest in this abortion modality following the *Dobbs* leak and echoing other research indicating increased online interest in medication abortion and use of SMA during this time [103-105]. Taken together, these findings suggest that, as legal access to abortion is increasingly constrained, people may be focusing less on *whether* to have an abortion and more on *how* to access it under legal constraints. The rise in discussions about SMA implies that more individuals may be pursuing abortion outside formal health care systems, often due to legal, logistical, or financial barriers, raising concerns about legal risks and equitable access to preferred, supported abortion care.

In the SMA conceptual group, posts covered a range of topics related to procuring and receiving medications, including concerns about the credibility of online ordering platforms. The topics in the SMA conceptual group align with previous research describing the experiences of using online platforms (often Aid Access), highlighting concerns about scams, ordering, shipping, and potential surveillance [106]. Additional work by our team expands these findings related to SMA, highlighting the many questions posted about accessing and using medications to self-manage and critical gaps in posters’ access to medical and legal information [107]. Notably, in the time since this study, the number of online platforms for ordering abortion medications has grown substantially, and abortion providers have started offering these medications legally from some states within the United States under shield laws [108]. As SMA becomes increasingly common and facilitated by more diverse pathways for obtaining medications, which is expected particularly in areas where abortion access is restricted [11,109], continued research is needed to understand emerging needs and challenges for people using online platforms to facilitate safe and satisfactory abortion experiences at home with limited clinical support. The experiences of people using the array of platforms facilitating access to medications for abortion are relatively unknown and warrant further research, particularly given likely differences in customer support and service quality across websites. In addition, efforts to protect and expand access to accurate online SMA information are critical as people rely on fully digital pathways to access services and face stigma, isolation, and legal risks that further limit access to information from nondigital sources.

This research explores abortion as a health and social concern on Reddit, with discussions influenced by the changing sociopolitical context in the United States*.* Previous qualitative research has explored how Reddit and r/abortion have been used for specific abortion-related concerns (eg, abortion costs and self-management barriers [28-33]), finding that analysis of Reddit data can provide meaningful insights into these concerns grounded in the unsolicited narratives shared in publicly available data. In addition, a recent study by Valdez et al [48] used NLP (specifically topic modeling with BERTopic) to describe discussions in a subset of posts to r/abortion and another Reddit community focused on abortion debate (r/AbortionDebate) and found that r/abortion was commonly used to seek and share social support, in contrast to r/AbortionDebate being used to discuss changing views on abortion. However, this analysis presents a novel approach to working with a theoretically complete sample of data from r/abortion during a period of sociopolitical changes that generated extreme uncertainty, fear, and constraint regarding abortion access in the United States. Our findings speak to the changing use of r/abortion during 2022 and the power of leveraging innovative research approaches grounded in content expertise to explore abortion as a critical public health concern.

### Strengths and Limitations

There are several important limitations to keep in mind when interpreting these findings*.* First, while using data from an abortion subreddit leverages the power of Reddit as a pseudonymous platform known to invite discussion of sensitive health concerns [24-26], we have no systematic demographic information about the r/abortion posters contributing to our sample. While most Reddit users across the entire platform are aged between 18 and 29 years (64%), male (64%), White individuals (70%), and based in the United States (52%) [110], research indicates that the demographics of members vary across subreddits, and in r/abortion, most users identify as female (86.1%) [111]. We also know that the population of Reddit and r/abortion users is unique [110,111] and likely does not represent people considering, seeking, and having abortions across the United States. As such, our findings cannot be interpreted as reflective of population-level experiences or concerns beyond r/abortion. Notably, in the post-*Dobbs* context, the access experiences of young people and those of a lower socioeconomic status are likely uniquely challenged as the impacts of *Dobbs* are experienced more intensely in these groups compared to others [1]. While the lack of demographic information limits our ability to determine who used r/abortion in 2022, there were stories shared in the community that represented a range of abortion experiences—including people who had not yet reached clinical abortion care, deciding between clinical abortion and SMA, and who never interacted with health care providers during their abortion processes. This representation of a diverse set of abortion experiences is a substantial contribution to our knowledge based on user-driven narratives and priorities.

Furthermore, although almost half of the users on Reddit live in the United States [20], it is a global platform. In qualitative analyses of a random subsample of these data, posts from people living outside of the United States were identified and excluded, accounting for 9% of the posts [37]. However, we took no similar steps for the current NLP analysis, introducing uncertainty about the connection of our results to the US abortion access context and policy change assuming that approximately 10% of all posts to r/abortion were from outside the United States. Despite this, we believe that US policy has a large enough impact on global perceptions of abortion access that this limitation does not substantially detract from our findings.

In addition, not all posts were correctly classified by BERTopic into topics, and perfect accuracy in classification is not expected. There are also methodological concerns related to our decision to use BERTopic model results to classify posts into a single topic as BERTopic operates under the assumption that documents can fall into multiple topics simultaneously. Choosing classification into a single topic allowed us to effectively make direct comparisons in the volume of posts per topic and conceptual group across subperiods but reduced consideration of the nuanced ways in which people talk about these topics within posts—often discussing multiple concerns that fall under different topics and perhaps different conceptual groups within 1 submission. It is plausible that this approach resulted in a substantial underestimation of the commonality of some topics within the corpus based on classification. However, in choosing to classify based on the topic with the highest probability for each post, we effectively captured the topics discussed most substantively in each submission—providing a simple and interpretable representation of the most dominant topic and related conceptual group described in each post.

Furthermore, while we attempted to be rigorous and precise in creating topic titles and aggregating topics into conceptual groups, the process was based on content expertise applied during subjective manual review guided by quantitative measures of topic similarity. Division of topics into conceptual groups was sometimes difficult, with posts in topics sometimes not exclusively describing clear concepts, underscoring concerns about using a single classification approach for each post. Delineating between topics related to medication abortion and SMA was conservative, erring on the side of only considering a topic as pertaining to SMA if it was very clearly focused on accessing, ordering, shipping, and receiving medications from an online platform. As such, this classification and aggregation approach likely underestimated the commonality of posts about SMA and perhaps the relative increase in those posts following the *Dobbs* leak and decision. This is further supported by the likelihood that people, despite the protection provided by pseudonymity on Reddit, may have limited their public sharing of information that overtly indicated that they were self-managing out of fear of prosecution [72,73].

Furthermore, the conceptualization of 3 subperiods in 2022 was based on key moments in national abortion policy but does not account for the rolling changes in abortion access within each of those periods. Even before the *Dobbs* leak, states were implementing abortion restrictions. Following the *Dobbs* decision, the enactment and enforcement of restrictions and bans across states has been progressive rather than the *Dobbs* decision functioning as a clear change point in all policies. As such, aggregating data into subperiods makes assumptions about the homogeneity of experiences of r/abortion users. Despite this concern, under the premise of exploring use of r/abortion and changes over time in the impacts of national abortion policy and related uncertainty about abortion access across states, using 3 subperiods as we did is a sound approach.

### Conclusions

Our analysis provides a holistic view of the content of post submissions to r/abortion in 2022. In this research, we were able to merge content expertise and machine learning tools to describe people’s posts to an online community for abortion during a time of extreme change and uncertainty in abortion access in the United States. Our findings highlight the critical role of r/abortion as an abortion-supportive resource, providing an online community for people to voice a vast array of concerns, questions, and experiences. They also illustrate how the use of r/abortion changed in 2022, speaking to the increased importance of SMA following the Dobbs leak. Overall, our findings highlight the need for further research exploring this trend across online platforms facilitating access to abortion information, support, and services—with particular focus on those providing access to abortion medications. As policies and pathways to abortion access continue to change across the United States, approaches leveraging NLP with sufficiently large samples of textual data present opportunities for timely monitoring, with the potential to reflect a broad range of abortion experiences, including those of people who have limited or no interaction with clinical abortion care.

## Appendix

Multimedia Appendix 1 Detailed processes for data acquisition and analyses.

Multimedia Appendix 2 Detailed results supplementing those in the manuscript text.

## Abbreviations

API: application programming interface
NLP: natural language processing
OARS: Online Abortion Resource Squad
SMA: self-managed abortion

## Data Availability

The datasets generated or analyzed during this study are available from the corresponding author on reasonable request.
