# Supplementary material for: Using Natural Language Processing to Describe the Use of an Online Community for Abortion During 2022: Dynamic Topic Modeling Analysis of Reddit Posts
Source: JMIR Infodemiology. 2025 Jul 9;5:e72771. doi: 10.2196/72771 (PMC12287674; doi:10.2196/72771)
Supplement: Multimedia Appendix 1 [file infodemiology_v5i1e72771_app1.docx]

**Appendix 1: Supplementary Methods**

***Data collection***

*Data collection approach 1 (12/24/2021-8/23/2022)*

For this research, data obtained from PushShift’s Reddit API were found to be incomplete, presenting substantial concerns for data quality related to PushShift’s scraping of data from Reddit at a single point in time (at least for past months of data [101]). This means that data from PushShift do not effectively reflect real-time updates to Reddit content, including interactions with posts (such as upvotes, downvotes, and comments) or changes in the status of posts (such as the content being removed or approved to be posted). For this research with data from r/abortion, this is a particular concern because many posts to this subreddit are automatically “removed” by spam and rule-based filters and later approved by moderators (and then posted). As such, the datasets our team obtained from PushShift’s Reddit API were missing the majority of the post text (noted as “[removed]” for post text, see Appendix 1 for by-period summaries of the proportions of removed and deleted content from PushShift). In contrast, data on the official Reddit API are updated in real-time [102], though this API has limitations on how content can be requested, not allowing data to be extracted by date but basing extraction on tags (e.g. “hot,” “top,” “new,” and “controversial”) and on the number of pulls per task (capped at 1000) [103].

Given the limitations of these two APIs, we used an approach combining information from both to obtain complete and current data from r/abortion. Specifically, we obtained monthly data for posts and comments from 12/24/2021-8/24/2022 using the Python Pushshift.io API Wrapper (PSAW, [104]) to interact with PushShift’s Reddit API, extracting “submission IDs” for each post and comment and saving these submission IDs as a list. These submission IDs, which are the same across the PushShift and Reddit APIs, were then used to pull complete data for the same period for each post and comment from Reddit’s API using the Python Reddit API Wrapper (PRAW, [105]). While the Reddit API provides information on a variety of indicators that offer opportunities for research on r/abortion, this research is focused specifically on the textual content of posts and comments, given the interest in exploring and learning from narratives of experience and support shared in this community. For submissions, the PRAW attributes scraped were: author, created_utc, is_self, link_flair_text, locked, name, num_comments, over_18, permalink, score, selftext, title, upvote_rate, url. For comments, the PRAW attributes scraped were: id, author, created_utc, parent_id, body, is_submitter, score, link_id, distinguished, edited, submission, stickied, replies. Post data for 12/24/2021-8/23/2022 was gathered and stored for cleaning and analysis on 16 October 2022; comment data for the same period on 18 November 2022.

*Data collection approach 2 (8/24/2022-12/24/2022)*

As of December 2022, PushShift’s Reddit API was no longer operational as a tool to access Reddit post data from prior to November 3, 2022 (only functional using the PMAW wrapper). But complete data could still be obtained from PushShift for comments using the PSAW wrapper. In PushShift data, comments are assigned a comments-specific submission ID number that contains the submission ID for the parent post. As such, the comment submission ID can be parsed to obtain unique post IDs for post submissions. We used this approach to obtain a list of unique post submission IDs from within the specified time period (8/23/2022-12/23/2022) from the r/abortion comments data obtained through PushShift. We then used these post IDs to pull complete data from the Reddit API using the same approach as described above. While this combined approach is not guaranteed to provide a complete list of unique post submission IDs, the r/abortion subreddit automatically responds to all posts with an ‘AutoModerator’ comment, ensuring that all posts should have at least one comment and therefore be included in the comments dataset. The primary missingness concern with this approach is the lack of complete information on removed and deleted content, as it is more likely to be excluded from the comments dataset. Comment submissions IDs obtained during this process were also used to pull complete comments data from the Reddit API using the same approach.

***Data pre-processing***

After procuring complete data from Reddit’s API, additional restrictions were implemented to obtain the analytic sample used for this research with specific exclusions for posts and comments. These restrictions were designed to provide a sample of posts theoretically within the public domain, containing sufficient text to support contextualized NLP analysis. Sequentially, posts were excluded if they:

1. Were “removed”
2. Were “deleted”
3. Contain only an image
4. Contains only a link
5. Contain <30 characters

This provided the analytic sample of posts. Comments responding to original posts were not included in this analysis. Content in this analytic sample was then cleaned to remove usernames, which were replaced with a unique submission ID specific to this research. This desensitized data was used for the analyses described. The broad sample of posts obtained provides a holistic view of the topics discussed by r/abortion community members in relation to their abortion-related questions, experiences, challenges, and more following the Dobbs decision and related changes in abortion access in the US in 2022.

To prepare our data for NLP analyses, data from posts were cleaned using the following steps:

- *Part of Speech tagging:* We used the python *nltk* library’s *pos_tag* to tokenize every post into words and tag them. We retained only singular nouns (‘NN’), singular proper nouns (‘NNP’), adjectives (‘JJ’), adverbs (‘RB’), verbs (‘VB’), and cardinal digits (‘CD’), helping in data dimensionality reduction.
- *Remove URLs/links:* We used the URLExtractor() Python library to detect links in any format. On inspection, we discovered that one caveat is that links enclosed within brackets were not getting removed, so we proceeded to:
  - First, remove the square and regular brackets, if any, from the data.
  - Next, replace all vertical tabs, if any, with spaces to split the dataset based on this character.
  - Finally, we use URLExtractor() to detect and remove all URLs from the dataset.
- *Remove Punctuation:* We used the Python string.punctuation to remove all punctuation from our data.
- *Change all data to lowercase.*
- *Remove words with two or fewer characters.*
- *Identify and remove stop words:* Stop words are articles, conjunctions, prepositions, pronouns and common verbs that, when removed, help the ML algorithm detect important content and improve the performance of the model. We used the Python nltk library English stopwords and extended it to include identified stopwords based upon iterations of exploratory data analysis.

### ***Analyses of cleaned post text***

#### Exploratory data analyses

We determined the frequency of word use within the cleaned text and then established distinctive words and phrases we used Python’s CountVectorizer set at max_df=0.95, min_df=0.00, and n-gram range [1,3] to fit and transform our data, generating top unigram, bigram, and trigram terms for each data frame (yearly and each study period). Min_df was set to 0 to account for the high diversity of words used across documents, which set no restriction for the minimum percentage of documents an n-gram had to appear in. Max_df was set to 0.95 to exclude any n-grams that appeared in more than 95% of all documents in the corpus. Next, the TF-IDF results and top 30 terms for each data frame were reviewed to guide the iterative data cleaning process, specifically, the designated stopwords excluded from our analyses. Final results were obtained to explore the distinctive words and phrases used across posts from the year and within three subperiods of 2022 of interest for this research: ‘pre-*Dobbs* leak’ (12/24/2021-5/01/2022), ‘*Dobbs* leak to decision’ (period from *Dobbs* leak to decision, 5/02/2022-6/23/2022), and ‘post-*Dobbs* decision’ (6/24/2022-12/23/2022).

### *Topic Modeling*

Topic models can be used to discover latent topics, or themes, in documents. For this analysis, each post was defined as a document for topic modeling, and each data frame represented a set of documents. BERTopic was used, given its capacity to account for the contexts of words in sentences in text, extending traditional topic modeling approaches that do not account for the semantic relationships between words [3]. It also has various opportunities to explore topic hierarchies, topic visualization, and topic analyses. We applied BERTopic with the n-gram range as (1,3) to conduct topic modeling. No other parameters were tuned (meaning they were set at default). Outlier documents were grouped into an outlier topic group (labeled as -1) in the modeling process and excluded from further review and analysis [4]. Topic numbers, labels (top 10 words), and associated documents were reviewed by the research assistant and lead researcher to assess model coherence. During this review process, a descriptive name was also developed and assigned for each topic based on the topic label and the raw text of representative submissions.

#### Document classification using topic models

Using the trained models, we categorized all r/abortion submissions using predictions in BERTopic [5]. Classification was done using raw text for submissions. While BERTopic generates topics under the assumption that documents can describe multiple topics, categorization was carried out to only assign each submission to the topic it had the highest probability of being in (rank 1 topic classification). This approach provided a single topic assignment for each post. Posts classified in each topic were reviewed to check the quality of assignments. Counts were obtained to ascertain the number of posts classified in each topic.

#### Topic Model Aggregation

Topic modeling was carried out inductively, yielding modeling results that we reviewed to determine a rigorous and consistent approach to topic aggregation. After reviewing topic labels and representative documents, it was clear that while many topics used distinctive words, they described similar concepts. As such, we were interested in aggregating topics into ‘conceptual groups,’ or clusters of related topics grouped into broader themes or domains that described key concepts represented by the topics in each model. Given the capacity of BERTopic to carry out the hierarchical aggregation of topics into reduced numbers of clusters, we were interested in leveraging this functionality. We used the *visualize_hierarchcy()* function with the topics generated using BERTopic to produce dendrogram visualizations of each model’s aggregation of topics based on the cosine distance matrix between topic embeddings. This visualization was used as a tool to understand quantitively assessed similarity across topics, as the dendrogram represents the clustering of topics.

When reviewing these hierarchical clustering results, we were aware that these topic model results might have limitations related to the unique nature of the language used in the dataset and the complexity of meaning in communication around abortion. As such, these visualizations were reviewed in combination with the documents classified in a topic for each topic model to determine if cosine similarity effectively captured conceptual similarities. During the review, it was clear that the hierarchical aggregation built into BERTopic accounted for much of the similarities across topics. However, it did not effectively manage instances where the language was similar but had different meanings within the context of pregnancy and abortion narratives (e.g., feel, test, nausea). Conceptual group assignments were manually determined for all topics using an approach that combined dendrogram results (quantitative) with a manual review of submission texts (qualitative), with a focus on defining conceptually meaningful and interpretable groups of topics. Post counts for each conceptual group were obtained based on the summed rank 1 classification assignments for topic modeling results, summarizing the commonality of submissions in each conceptual group for each data frame. These aggregated topic groups were combined with the submission classifications.

#### Assessing changes in conceptual group frequency over time

Counts of submissions in each conceptual group for the year were used to assess the proportion of posts in each group and differences between the subperiods of interest (‘pre-*Dobbs* leak’, ‘*Dobbs* leak to decision’, and ‘post-*Dobbs* decision’). Statistically significant differences in the proportion of posts in a conceptual group were assessed using chi-squared tests in R (chisq.test). This compared the proportion of posts in a conceptual group vs. not in that conceptual group across the three study sub-periods, assessing if there was a change in the frequency of posts primarily focus on that concept over time. This component of the analysis seeks to describe any changes in the primary focus of r/abortion submissions during 2022, considering the dramatic changes to the legal and social environment for abortion during the year.

***References***

[1] K. Sparck Jones, “A Statistical Interpretation of Term Specificity and its Application in Retrieval,” *Journal of Documentation*, vol. 28, no. 1, pp. 11–21, Jan. 1972, doi: 10.1108/eb026526.

[2] C. Ren and I. Bloemraad, “New Methods and the Study of Vulnerable Groups: Using Machine Learning to Identify Immigrant-Oriented Nonprofit Organizations,” *Socius*, vol. 8, p. 23780231221076992, Jan. 2022, doi: 10.1177/23780231221076992.

[3] M. Grootendorst, “BERTopic: Neural topic modeling with a class-based TF-IDF procedure,” Mar. 11, 2022, *arXiv*: arXiv:2203.05794. Accessed: Jun. 29, 2023. [Online]. Available: http://arxiv.org/abs/2203.05794

[4] M. P. Grootendorst, “Quick Start - BERTopic.” Accessed: Jul. 17, 2023. [Online]. Available: https://maartengr.github.io/BERTopic/getting_started/quickstart/quickstart.html

[5] M. P. Grootendorst, “BERTopic - BERTopic.” Accessed: Apr. 05, 2024. [Online]. Available: https://maartengr.github.io/BERTopic/api/bertopic.html
