# Supplementary material for: Using Natural Language Processing to Describe the Use of an Online Community for Abortion During 2022: Dynamic Topic Modeling Analysis of Reddit Posts
Source: JMIR Infodemiology. 2025 Jul 9;5:e72771. doi: 10.2196/72771 (PMC12287674; doi:10.2196/72771)
Supplement: Multimedia Appendix 2 [file infodemiology_v5i1e72771_app2.docx]

**Appendix 2: Supplementary Results**

**Table A2.1:** Distinguishing uni-, bi-, and trigram results for posts by study period (N=7273 posts).

| **N-gram rank^a^** | **Before the Dobbs leak (December 24, 2021-May 1, 2022; n=2053)** | | | **Dobbs leak to decision (May 2, 2022-June 23, 2022; n=995)** | | | **After the Dobbs decision (June 24, 2022-December 23, 2022; n=4225)** | | | **Year overall (December 24, 2021-December 23, 2022)** | | |
| --- | --- | --- | --- | --- | --- | --- | --- | --- | --- | --- | --- | --- |
|  | ***Unigram (frequency)^b^*** | ***Bigram (frequency)*** | ***Trigram (frequency)*** | ***Unigram (frequency)*** | ***Bigram (frequency)*** | ***Trigram (frequency)*** | ***Unigram (frequency)*** | ***Bigram (frequency)*** | ***Trigram (frequency)*** | ***Unigram (frequency)*** | ***Bigram (frequency)*** | ***Trigram (frequency)*** |
|  | | | | | | | | | | | | |
| **1** | “Christmas” (n=33) | “Extremely nervous” (n=13) | “Pain ever experienced” (n=12) | “Texan” (n=14) | “Texan mom” (n=10) | “Roe wade overturned” (n=5) | “Christmas” (n=34) | “Red state” (n=47) | “Live red state” (n=26) | “Abortion” (n=6976) | “Red state” (n=47) | “Live red state” (n=26) |
| **2** | “Responded” (n=16) | “Kids already” (n=13) | “Still testing positive” (n=9) | “Overturned” (n=8) | “Days stopped” (n=7) | “People get sick” (n=4) | “Overturned” (n=34) | “Mife miso” (n=27) | “Live state abortion” (n=21) | “Feel” (n=5768) | “Medical abortion” (n=597) | “Took pregnancy test” (n=165) |
| **3** | “Play” (n=15) | “Cramps felt” (n=12) | “Pregnancy symptoms went” (n=8) | “Dot” (n=6) | “May took” (n=6) | “Found pregnant may” (n=4) | “Play” (n=29) | “Live red” (n=26) | “Roe wade overturned” (n=19) | “Know” (n=5383) | “Birth control” (n=580) | “Second set pills” (n=75) |
| **4** | “Wheeled” (n=15) | “Check pad” (n=12) | “Weeks ago still” (n=8) | “Fantasy” (n=6) | “May weeks” (n=6) | “Two positive pregnancy” (n=4) | “Gives” (n=28) | “Illegal state” (n=21) | “Still testing positive” (n=16) | “Weeks” (n=5091) | “Pregnancy test” (n=565) | “Pills aid access” (n=71) |
| **5** | “Gives” (n=14) | “Felt amazing” (n=11) | “Made feel comfortable” (n=8) | “Messaging” (n=5) | “Step mom” (n=6) | “First pill got” (n=4) | “Packaging” (n=26) | “Wade overturned” (n=20) | “Small blood clots” (n=15) | “Pregnant” (n=4407) | “Weeks pregnant” (n=557) | “Abortion weeks ago” (n=63) |
| **6** | “Nature” (n=13) | “Process abortion” (n=11) | “Sent back waiting” (n=7) | “Coparent” (n=5) | “Also currently” (n=6) | “Four tablets misoprostol” (n=4) | “Theres” (n=24) | “Extremely nervous” (n=19) | “Advice greatly appreciated” (n=15) | “Want” (n=4208) | “Planned parenthood” (n=499) | “Medical abortion weeks” (n=56) |
| **7** | “Favorite” (n=12) | “Swallowed rest” (n=11) | “Medical pill abortion” (n=7) | “Backed” (n=4) | “Today may” (n=6) | “Let everyone know” (n=4) | “Yeast” (n=22) | “Round pills” (n=19) | “State abortion illegal” (n=15) | “Time” (n=4169) | “Weeks ago” (n=432) | “Know right decision” (n=56) |
| **8** | “Siblings” (n=12) | “Pills mail” (n=11) | “Symptoms went away” (n=7) | “Nemo” (n=4) | “Ashamed texan” (n=6) | “Days stopped bleeding” (n=4) | “Approved” (n=21) | “Legal state” (n=19) | “State abortion legal” (n=12) | “Took” (n=3762) | “Surgical abortion” (n=420) | “Wanted share experience” (n=55) |
| **9** | “Woken” (n=12) | “Feel completely” (n=11) | “Found pregnant december” (n=7) | “Stigmatism” (n=4) | “Buy pregnancy” (n=5) | “Helped get dressed” (n=4) | “Damage” (n=21) | “Miso took” (n=18) | “Last night took” (n=12) | “Pregnancy” (n=3556) | “Got pregnant” (n=380) | “Long story short” (n=52) |
| **10** | “Respectful” (n=12) | “Sure okay” (n=10) | “Home pregnancy tests” (n=6) | “Season” (n=4) | “Currently living” (n=5) | “Buy pregnancy test” (n=4) | “Boys” (n=20) | “Small blood” (n=18) | “Weeks pregnant took” (n=12) | “Bleeding” (n=3529) | “Days ago” (n=364) | “Weeks days pregnant” (n=51) |
| **11** | “Excessive” (n=11) | “Never expected” (n=10) | “Make sure okay” (n=6) | “Emetophobia” (n=4) | “Quarter sized” (n=5) | “Worst part whole” (n=4) | “Hg” (n=20) | “Days feel” (n=18) | “Post preparing shipment” (n=11) | “Really” (n=3433) | “Weeks days” (n=345) | “Know right choice” (n=49) |
| **12** | “Preparation” (n=11) | “New pregnancy” (n=10) | “Gotten period yet” (n=6) | “Zs” (n=4) | “Taking responsibility” (n=5) | “Hour half away” (n=4) | “Sites” (n=20) | “Normal anyone” (n=18) | “First dose miso” (n=11) | “Pain” (n=3267) | “Took pill” (n=334) | “Positive pregnancy test” (n=46) |
| **13** | “Coil” (n=11) | “Symptoms went” (n=10) | “Ago still bleeding” (n=6) | “Norco” (n=4) | “Dull ache” (n=5) | “Weeks took pregnancy” (n=4) | “Wine” (n=19) | “Lemon sized” (n=18) | “Origin post preparing” (n=11) | “Day” (n=3236) | “Make sure” (n=306) | “Took miso pills” (n=46) |
| **14** | “Tub” (n=10) | “Mins later” (n=10) | “Found weeks days” (n=6) | “Mile” (n=4) | “Wade overturned” (n=5) | “First abortion pill” (n=4) | “Failing” (n=18) | “Update took” (n=17) | “Almost weeks ago” (n=11) | “Got” (n=3022) | “Heating pad” (n=290) | “Appointment planned parenthood” (n=45) |
| **15** | “Sites” (n=10) | “Consent form” (n=9) | “Couple days late” (n=6) | “Practitioner” (n=4) | “Back february” (n=5) | “Bleeding anything hours” (n=3) | “Responded” (n=18) | “Days work” (n=17) | “Bed heating pad” (n=10) | “Days” (n=3002) | “Took pills” (n=269) | “Hot water bottle” (n=43) |
| **16** | “Labour” (n=10) | “Toilet felt” (n=9) | “Get pregnancy test” (n=6) | “Groin” (n=4) | “Five days” (n=5) | “Abortion last night” (n=3) | “Remains” (n=18) | “Miso hours” (n=17) | “Pregnant week ago” (n=10) | “Period” (n=2959) | “Abortion pills” (n=267) | “Pregnant weeks ago” (n=42) |
| **17** | “Board” (n=10) | “Procedure also” (n=9) | “Around weeks pregnant” (n=6) | “Overseas” (n=4) | “Abortion april” (n=5) | “Bed watching movie” (n=3) | “Preg” (n=18) | “Cramps felt” (n=17) | “Take pain meds” (n=10) | “Pills” (n=2894) | “Abortion weeks” (n=252) | “Surgical abortion weeks” (n=42) |
| **18** | “Msi” (n=10) | “Abortion successful” (n=9) | “Night next day” (n=6) | “Anxieties” (n=4) | “Suspected pregnant” (n=5) | “Weeks taking first” (n=3) | “Affecting” (n=18) | “Pills tomorrow” (n=16) | “First pill clinic” (n=10) | “Felt” (n=2752) | “Started bleeding” (n=244) | “Birth control pills” (n=39) |
| **19** | “Voices” (n=10) | “Day time” (n=9) | “Decision ever made” (n=6) | “Tylonel” (n=4) | “Hours bleed” (n=4) | “Bleeding fresh blood” (n=3) | “Favorite” (n=17) | “Part wants” (n=16) | “Went back sleep” (n=10) | “Going” (n=2747) | “Blood clots” (n=235) | “Bring child world” (n=37) |
| **20** | “Dilapan” (n=10) | “New year” (n=9) | “Trying get pregnant” (n=6) | “Restaurant” (n=4) | “Sa may” (n=4) | “Day yesterday bleeding” (n=3) | “Leg” (n=17) | “Back sleep” (n=16) | “Second round pills” (n=10) | “Went” (n=2557) | “Hours later” (n=229) | “Anti nausea meds” (n=37) |
| **21** | “Weather” (n=10) | “Baby still” (n=9) | “Wanted say thank” (n=6) | “Dupont” (n=4) | “Locate fetus” (n=4) | “Weeks days post” (n=3) | “Fainting” (n=17) | “Advice greatly” (n=16) | “Bleeding passing clots” (n=10) | “Baby” (n=2490) | “Pregnancy symptoms” (n=219) | “Went planned parenthood” (n=37) |
| **22** | “Hateful” (n=9) | “Successful abortion” (n=9) | “Long term relationship” (n=6) | “Midwifery” (n=4) | “Experience felt” (n=4) | “Found pregnant april” (n=3) | “Folks” (n=16) | “Period july” (n=16) | “Passed small clots” (n=10) | “Right” (n=2374) | “Want baby” (n=213) | “Took pill mifepristone” (n=36) |
| **23** | “Episodes” (n=9) | “Want someone” (n=9) | “First pill clinic” (n=6) | “Od” (n=4) | “Sugar pill” (n=4) | “Anything help pain” (n=3) | “Preparation” (n=16) | “Look back” (n=16) | “Stay home mom” (n=10) | “Week” (n=2180) | “Mental health” (n=210) | “Pregnancy test weeks” (n=36) |
| **24** | “Bathtub” (n=9) | “Worst thing” (n=9) | “Recovery room nurse” (n=6) | “Thereafter” (n=4) | “Forward midwifery” (n=4) | “Bleeding still bleeding” (n=3) | “Fl” (n=16) | “Pills cheeks” (n=15) | “Hi surgical abortion” (n=10) | “Told” (n=2166) | “Really want” (n=205) | “Hours later took” (n=36) |
| **25** | “Nupas” (n=9) | “Still blood” (n=8) | “Waiting room waiting” (n=6) | “Locate” (n=4) | “Tomorrow procedure” (n=4) | “Experience planned parenthood” (n=3) | “Ohio” (n=16) | “Breaks heart” (n=15) | “Go back time” (n=10) | “Think” (n=2139) | “Getting abortion” (n=198) | “Abortion years ago” (n=35) |
| **26** | “Hpt” (n=8) | “People saying” (n=8) | “Inserted misoprostol vaginally” (n=6) | “Doorstep” (n=4) | “Pills may” (n=4) | “Make sure passed” (n=3) | “Unit” (n=16) | “Baby really” (n=15) | “Normal anyone else” (n=10) | “Hours” (n=2048) | “Took pregnancy” (n=197) | “Test came positive” (n=34) |
| **27** | “Shoulders” (n=8) | “Reading people” (n=8) | “Past couple weeks” (n=6) | “Fading” (n=3) | “Medication working” (n=4) | “Later inserted tablets” (n=3) | “Fredli” (n=15) | “Abortion november” (n=15) | “Last night around” (n=9) | “Life” (n=1920) | “Abortion pill” (n=193) | “Abortion days ago” (n=34) |
| **28** | “Suctioning” (n=8) | “Night went” (n=8) | “Weeks pregnant took” (n=6) | “Hoops” (n=3) | “Next wednesday” (n=4) | “Planned parenthood really” (n=3) | “Remainder” (n=15) | “Period coming” (n=15) | “Almost two months” (n=9) | “Help” (n=1892) | “Waiting room” (n=189) | “Took second dose” (n=34) |
| **29** | “Prep” (n=8) | “Feel deserve” (n=8) | “Pills hours ago” (n=5) | “Tore” (n=3) | “Time way” (n=4) | “Insert misoprostol pills” (n=3) | “Chalky” (n=15) | “Go see” (n=15) | “Test days ago” (n=9) | “Blood” (n=1854) | “Know right” (n=189) | “Want make sure” (n=34) |
| **30** | “Portion” (n=8) | “Feeling going” (n=8) | “Heating pad belly” (n=5) | “Intensive” (n=3) | “Track periods” (n=4) | “Anything hours later” (n=3) | “Market” (n=15) | “Bleeding small” (n=15) | “Someone please tell” (n=9) | “Wanted” (n=1846) | “Period cramps” (n=186) | “Know right thing” (n=34) |

^a^*N-gram* refers to a sequence of *n* words that appear next to each other in text (eg, a 2-word phrase is called a *bigram*). Ranks are based on how often an n-gram appears compared to others—rank 1 being the most common single-, 2-, or 3-word phrase in the analyzed body of text.

^b^N-gram frequency is the number of times that an n-gram occurs in the body of tex

**Table A2.2: Composite quotes for select topics in each yearly conceptual group**

| **Conceptual group** | **Topic** | **Composite quote** |
| --- | --- | --- |
| Navigating access barriers | Navigating access barriers, accessing in-clinic care | *“My boyfriend and I live in the south with our 9 month old baby, and we just found out I'm pregnant again. We really can't afford to and aren't prepared to have another child. Abortion is illegal in our state and we live 4 hours from the nearest clinic in the nearest legal state. Do we have any other options?”* |
|  | Navigating access barriers, seeking access to medication abortion navigating barriers | *“My girlfriend and I are in urgent need of abortion pills. She's about 3 or 4 weeks pregnant and we want to get through this asap and for not a lot of money, preferably for a few hundred dollars or less. Can anyone provide some info and walk us through the process?”* |
|  | Navigating access barriers, Dobbs decision | *“I think I might be pregnant and I'm really scared, especially after hearing Roe v Wade might be overturned. I'm thinking of having an abortion but I live in a state where abortion will likely become illegal right away. Does anyone know if I will still be able to get an abortion somehow? I've been trying to research online and have been seen a lot of different information. Are there any online clinics? Would it be illegal to travel to another state to go to a clinic in-person? I've heard that Colorado or Illinois might be safe options but I'm just really terrified. I just want to be sure I can access an abortion no matter what happens.”* |
| Medication abortion process | Medication abortion process, timing of pills | *“I ordered pills through AidAccess and they arrived today. I've seen online that you're supposed to wait 24 hours after you take the mifepristone to then take the misoprostol, but on the box it doesn't mention timing and the instructions are very vague. I don't want to screw this up at all so can someone clarify the timing and what exactly I should do?”* |
|  | Medication abortion process, seeking information about access and use experience | *“I have an appointment at Planned Parenthood later this week. I had a surgical abortion a few years ago at 12 weeks. My period is hard to track, but I think I'm between 4 and 6 weeks, so I think I can still get the pill. What is the pill like? My surgical abortion was really intense and I'm thinking the pill might be easier. What should I know about the process? Do I need to take time off of work? What symptoms should I know about? I want to be prepared. This community makes me feel less alone! Thank you!”* |
| Abortion decision-making | Abortion decision-making, sharing stories and seeking support | *“Can anyone share about their experiences during and after their abortions around 6 weeks? I'm trying to pick between medication or surgical. My last abortion was surgical and it was not a great experience. I just want this to be over with because ideally, I wouldn't want to have to go through this, but I am not in a place to support a child financially.”* |
|  | Abortion decision-making relationship dynamics | *“I can't take this anymore, I'm going to get an abortion. I'm just not ready to have a baby and I know that this will be for the best. I'm not looking forward to having to face my partner after this. I'm afraid he's going to hate me for having an abortion and break up with me.”* |
|  | Abortion decision-making support (presence and lack) | *“I'm feeling a lot emotions after my abortion. I just feel so guilty and undeserving of love right now. I noticed that my support system is unsure of how to comfort me, so I'd rather just be alone than to make them feel bad. I just need some space. This subreddit has been helping me get through it all.”* |
| Post-abortion physical experiences | Post-SA bleeding | *“I had a surgical abortion last week and have had some bleeding like a period since then. I've also had some mild cramping. Is it normal to bleed a little? When can I expect it to stop?”* |
|  | Post-abortion clots | *“I took my miso last night and have been bleeding for almost 12 hours now. I had cramping for the first few hours then a big flow of blood where I passed two big clots the size of small lemons. But those are the only clots I've had and I haven't been bleeding any more. I know you're supposed to pass a lot of clots based on what I've been reading. Should I be worried?”* |
| Clinical abortion care | Clinical abortion care, fears | *“I am 21 years old and I want to get an abortion. I know this is the right decision for me, but I'm still feeling really terrified, as well as some feelings of grief and sadness. I'm having the abortion in a clinic in a few days but I just want this to be over.”* |
|  | Clinical abortion care, ultrasound | *“I'm traveling to a different state to have an abortion and I need to get an ultrasound. I don't know if I should get one in my home state because abortion is illegal where I live and I don't want anyone to find out I'm pregnant. Will the state find out if I do get one? Should I go somewhere else? I don't even know how to get this done and I need help.”* |
| Potential pregnancy | Pregnancy risk and sex | *“My period is almost a week late and it has never been late before. I had unprotected sex but he didn't finish inside of me. I'm pretty sure I was ovulating. Am I pregnant? Please help!”* |
|  | Potential pregnancy testing | *“I had an abortion over a month ago. I took a pregnancy test last week and again this week and they were both positive. Could I be pregnant again? How long do people usually test positive after? I'm so worried with what's happening with the supreme court right now.”* |
| SMA processes | Aid Access ordering | *“Has anyone used Aid Access and honeybee? I had a consultation and sent in a picture of my ID. How long does it take for them to get back to you? I'm using them because I don't have much money and need an abortion.”* |
|  | Aid Access shipping | *“There have been a lot of posts about Aid Access on here, so I want to share my experience. I contacted Aid Access almost three weeks ago. I sent my payment a few days later, then they followed up within a day to confirm my information and send my prescription update. I got am email the day after saying that my shipment was being prepared. My package got to customs within a few days, then to the shipping facility almost a week later. It got to my state (where abortion is not legal) two days later, then was delivered to me yesterday. Hopefully this helps someone else going through this process.”* |

**Table A2.3: Composite quotes for selected topics in each pre-*Dobbs* leak conceptual group**

| **Conceptual group** | **Topic** | **Composite quote** |
| --- | --- | --- |
| Abortion decision-making | Abortion decision making, navigating challenges | *“I've been reading posts on here since the day I found out I was pregnant. Thank you to everyone who's shared information and been kind. My period was late by a few days so I took a pregnancy and it was positive. I took two days figuring out what I wanted to do. I'm in my 30s and have always wanted to have kids. But I don't have a good relationship with the father and am not in a good place to raise a kid. I still want to be a mom, but was not excited to be pregnancy and start being a mother right now. I have my abortion last week and do not regret it. I feel relieved that my body will be normal again soon. But I am sad, just because I had an abortion doesn't mean I don't want children.”* |
|  | Abortion decision-making sharing feelings and stories | *“My due date would have been later this week. I have a two year old and got pregnant by accident. I decided to abortion for a lot of reasons and the pregnancy was very stressful for me. I wasn't sleeping or eating. I couldn't work because I was so anxious. And after my abortion I was so relieved, my anxiety was gone. I know it was for the best, but I still feel sad when I see other people who are pregnant or have babies. It doesn't make sense. Has anyone else felt this way?”* |
| Post-abortion physical experiences | Post-abortion bleeding and pain | *“I had a surgical abortion at 9 weeks four days ago. The procedure was really terrible. I was bleeding and having some cramping for a couple of days. Now I'm not having any cramping or bleeding at all. I'm worried this means it didn't work.”* |
|  | Post-abortion understanding 'normal' physical experiences | *“Last week I had an abortion at 15 weeks. Today I passed a huge clot, like the size of two fists. I keep having cramps and bleeding. Is this normal?* |
| Navigating access barriers | Seeking abortion access, uncertainty and restrictions | *“I'm 21 and just found out I'm pregnant. I'm too young to have a kid and don't want the responsibility. I want the abortion pill and am curious what everyone's experiences have been. I live in a state where abortion is illegal, so I'm not sure the best way to get it. How does the process go? I want to hear people's stories to feel better about the process.”* |
| Medication abortion process | Medication abortion physical process, mifepristone and misoprostol | *“I was around 7 weeks when I took my misoprostol yesterday. For the first few hours I was having cramps then I had the worst pain. I went in the bathroom and passed a huge clot. The pain kept getting worse, then I started having diarrhea. I couldn't see what I was passing because of everything else coming out of me. I think I was passing clots because there was blood in the water. The pain continued for another hour then when I went to wipe, I think I wiped the sac because there was a pink white blob. My pain was 8 out of 10.”* |
|  | Medication abortion physical process, pain and bleeding | *“I took my pills a few hours ago and now I'm in a lot of pain. I started bleeding and am having cramps that make me feel like I'm going to vomit. This is the worst I've ever felt, even though I took ibuprofen before like they told me to. I can't even move it hurts so much but trying to hold on. What helped others who felt the same?”* |
| Clinical abortion care | Clinical abortion care, fears and pain | *“I'm having a surgical abortion in a few days. I'm going to be put under but I'm scared. I'm really nervous about the whole thing. What if I feel it? Or what if I don't wake up? Any support from people who've also done this would be helpful. I just want this to over!”* |
|  | Seeking medication abortion access in-clinic | *“I have an appointment to get a MA and am not sure what to expect. How long will I be at the clinic? My appointment is at 9 am. I have that day and the next one off of work. Is it ok to go to work within a day after taking my second set of pills?”* |
| Potential pregnancy | Potential pregnancy and abortion access | *“My girlfriend and I had sex with a condom two weeks ago. She said her period is 5 days late. We're freaking out. Can anyone help? We live in a red state and would need consent from our parents to have an abortion and I don't think that's an option. What should we do?”* |
|  | Potential pregnancy and testing | *“I had a surgical abortion a month ago. I had unprotected sex two weeks ago. The clinic said I wouldn't have a period for a month or two, but I'm worried I'm pregnant. I woke up throwing up but when I did a pregnancy test it was negative. Could I still be pregnant? Do I need to keep taking tests to know for sure?”* |

**Table A2.4: Composite quotes for select topics in each *Dobbs* leak to *Dobbs* decision conceptual group**

| **Conceptual group** | **Topic** | **Composite quote** |
| --- | --- | --- |
| Clinical abortion care | Clinically supported abortion processes | *“I've seen a lot of posts about people having really bad experiences with surgical abortions. Are they getting sedation or numbing? I don't want to be in pain and I'm really worried. I have an appointment for next week.”* |
| Navigating access barriers | Navigating uncertainty and restrictions | *“I'm pregnant and need an abortion. I'm very certain about my decision. My boyfriend and mom know and support me. I had an appointment and then realized it was with some faith organization that's against abortion, Planned Parenthood doesn't do abortions where I live. All of the other clinics I called haven't been helpful. I don't know what to look for. Please help.”* |
| Abortion experiences | Intra abortion physical experiences, bleeding and pain | *“I'm sharing my story to help other people like this forum helped me. I was almost 10 weeks, no money and needing to get an abortion fast. I ordered the kit online and it came in less than a week. For people doing this, you should make sure to have pads and be patient. Also be ready for what you could see. I took the first pill, then the other 4 12 hours later. I had a lot of cramping but no pain. I was worried it wasn't working, but then after hours the fetus came out. I felt sad and relieved at the same time. I didn't want to just flush it down the toilet. Wishing everyone else the best in the process.”* |
| Post-abortion physical experiences | Post-abortion bleeding and return to menstruation | *“I was bleeding for weeks after my abortion then it stopped for a few days and I was finally feeling normal. I even had sex with my partner. Then the bleeding started again. Is that normal?”* |
| Potential pregnancy | Potential pregnancy and pregnancy tests | *“I had a surgical abortion 6 weeks ago. I took a pregnancy test and it was positive. Is that normal? I read some posts that said that you can get a false positive because of the hormones. But I'm nervous the abortion didn't work. I did have protected sex last week, but I doubt that's causing it since I don't have any pregnancy symptoms. I'm really stressed.”* |
| SMA process | SMA ordering and shipping | *“Has anyone used onlineaboritonpillsrx to order pills? Their site looks suspicious, but I need to get pill soon. They're almost $500 and I'm worried about paying that much and also paying for overnight shipping. But AidAccess will probably take too long. Also what does the packaging look like from their site? I live with my parents and they can't find out.”* |
| Abortion decision-making | Abortion decision-making, timing and wantedness | *“I want to have this kid, but the more I think about it the more I'm sure I can't have it. I never wanted to have one because I didn't think I could handle it. And it's already too much. I can't do it and I feel like a failure. I can't talk to anyone about this.”* |

**Table A2.5: Composite quotes for select topics in each post-*Dobbs* leak conceptual group**

| **Conceptual group** | **Topic** | **Composite quote** |
| --- | --- | --- |
| Navigating access barriers | Seeking access to medication abortion in restrictive settings | *“Where I live, abortion is banned after 6 weeks. I'm thinking about traveling to another state to get pills. I would like to fly, but am worried about having pills in my carry on bag. Any suggestions?”* |
|  | Navigating structural barriers | *“Do I need to have an ID to get an abortion? I don't have my license or real ID yet, but would other forms of ID work like a school ID or birth certificate?”* |
| Medication abortion process | Medication abortion process, bleeding, cramping, and passing clots | *“It's been almost a week since my MA and I'm having really bad cramps. I'm also still bleeding. I don't have a fever or anything else. Is this normal? I called the hotline and they said it's normal but it doesn't feel that way.”* |
|  | Medication abortion process, timing of experiences with pills | *“I'm just over 7 weeks. I took my first pill at the clinic this morning around 10 and the nurse told me to take my miso vaginally right after if I wanted. So I inserted 8 miso at noon and am having horrible cramps and heavy bleeding with clumps. I'm worried it's not working! Has anyone experienced something similar?”* |
| Potential pregnancy | Post-abortion completion and pregnancy tests | *“Abortion is banned where I live and I recently had a medical abortion. During an appointment, my doctor told me that I could be pregnant, but I took multiple at-home tests and they were all negative. What does it mean? Could it be that the abortion wasn't complete and what should I do?”* |
|  | Potential pregnancy, sexual risk | *“I'm worried that I'm pregnant because my period is late. I'm sexually active, but I think I was safe. How many days should I wait to take a pregnancy test or can I take one now?”* |
| Post abortion physical experiences | Post-abortion feelings, coping and seeking support | *“Recently had a MA and I didn't feel anything before, it was just something I needed to do. But today, I'm just experiencing a lot of emotions after expelling the tissue. It's probably hormones, but wondering if anyone else felt like this?”* |
|  | Post-abortion bleeding and clots | *“I had a medical abortion around 2 months ago and everything seemed okay until I started having clots and not from my period. I'm noticing large clots almost every hour or few hours. It'll almost be a week and I wanted to know if this is usual?”* |
|  | Post-abortion bleeding and menstruation | *“After my MA, I bled for a few weeks until it lessened into spotting. I feel like I'm not pregnant anymore, but the bleeding just came back and now I'm worried if this is right? Has anyone also had their period cycle messed up after abortion?”* |
| Clinical abortion care | In-clinic abortion procedures | *“I have a surgical abortion scheduled for next week and it feels so far away. My morning sickness is so bad and I can't get anything done. I was going to have an MA but decided to get a the procedure but I don't know what to expect. To anyone who has done an SA, what was the hardest part? Is it painful? Do you remember the procedure? How long until I go back to normal after?”* |
|  | SA process, anxiety | *“I have my surgical abortion tomorrow and I'm really nervous. I'm really scared about what it will be like after, because I know the procedure will be quick. But I feel like the bleeding, pain, cramps, and everything are punishment after already going through all of this.”* |
| Abortion decision-making | Abortion decision-making timing and feelings | *“I'm not ready to have a child. My boyfriend isn't ready to have a child. We both have a lot we want to do so that we can be ready one day. I know having an abortion was the right decision for me, even though I have been feeling broken. But I know we're heading towards something better.”* |
| SMA process | Online medication abortion ordering shipping, tracking, delivery | *“My Aid Access package has been in customs for over a week. Has this happened to anyone else? And if it has, did you get your package? It's been almost two weeks since I ordered and I'm worried I need to look for another option to get my pills on time.”* |
|  | Online medication abortion ordering credibility and payments | *“I'm looking for the pill and saw people posting about Aid Access on here. It seems like they've helped a lot of people but I'm skeptical. It seems too easy. I'm worried I'm going to get scammed or hacked. They asked me to send payment to an email. Has anyone else had this experience? How long did it take for you to get pills?”* |
|  | Online medication abortion ordering process and timing | *“I ordered pills from Aid Access, but they haven't arrived. I keep checking the order status, but it's been at the same location for a while. I really need them soon, how long did it take for everyone else to receive their pills? And is there another place I can order from if they don't come?”* |

**Table A2.6: Counts and proportions of posts in each conceptual group for the year and by study period with comparisons of proportion in each group across periods**

| **Conceptual group** | **Posts, year**  **(n=7,270)** | | **Posts, before *Dobbs* leak^a^**  **(n=2,052)** | | **Posts, *Dobbs* leak to decision**  **(n=993)** | | **Posts, after *Dobbs* decision**  **(n=4,225)** | | **Chi-squared value** | **p-value** |
| --- | --- | --- | --- | --- | --- | --- | --- | --- | --- | --- |
|  | **N** | **%** | N | % | N | % | N | % |  |  |
| Navigating access barriers | **2446** | **33.87%** | 677 | 27.68% | 346 | 14.15% | 1423 | 58.18% | 1.03 | 0.60 |
| Medication abortion process | **1807** | **24.86%** | 491 | 27.17% | 230 | 12.73% | 1086 | 60.10% | 4.10 | 0.13 |
| Abortion decision-making | **974** | **13.49%** | 321 | 32.96% | 129 | 13.24% | 524 | 53.80% | 12.67 | **0.002** |
| Post-abortion physical experiences | **672** | **9.30%** | 187 | 27.83% | 88 | 13.10% | 397 | 59.08% | 0.33 | 0.85 |
| Clinical abortion care | **670** | **9.28%** | 200 | 29.85% | 102 | 15.22% | 368 | 54.93% | 3.31 | 0.19 |
| Potential pregnancy | **550** | **7.62%** | 156 | 28.36% | 80 | 14.55% | 314 | 57.09% | 0.45 | 0.80 |
| Self-managed abortion process^b^ | **151** | **2.09%** | 20 | 13.25% | 18 | 11.92% | 113 | 74.83% | 20.02 | **<0.0001** |

^a^ Classification counts for posts in each conceptual group are based on yearly topic modeling results, with groups obtained from yearly model results used to classify to posts from each subperiod.

^b^ Self-managed abortion (SMA) is defined for this research as taking action to end a pregnancy outside of the formal healthcare system with or without clinical support, which includes the use of safe medications such as misoprostol and mifepristone but also potentially harmful or ineffective methods [82].
